# A Distributed Strategy for Cooperative Autonomous Robots Using Pedestrian Behavior for Multi-Target Search in the Unknown Environment

**DOI:** 10.3390/s20061606

**Published:** 2020-03-13

**Authors:** Haiyun Shi, Jie Li, Zhi Li

**Affiliations:** 1 College of Electronics and Information Engineering, Sichuan University, 610065 Chengdu, China; haiyunscu@163.com; 2 The Institute of Computer Science, The Beijing University of Posts and Telecommunications, 100876 Beijing, China; jli@bupt.edu.cn; 3 Key Laboratory of Wireless Power Transmission of Ministry of Education, Sichuan University, 610065 Chengdu, China

**Keywords:** distributed strategy, pedestrian behavior, swarm intelligence, swarm robots, multi-target search

## Abstract

Searching multiple targets with swarm robots is a realistic and significant problem. The goal is to search the targets in the minimum time while avoiding collisions with other robots. In this paper, inspired by pedestrian behavior, swarm robotic pedestrian behavior (SRPB) was proposed. It considered many realistic constraints in the multi-target search problem, including limited communication range, limited working time, unknown sources, unknown extrema, the arbitrary initial location of robots, non-oriented search, and no central coordination. The performance of different cooperative strategies was evaluated in terms of average time to find the first, the half, and the last source, the number of located sources and the collision rate. Several experiments with different target signals, fixed initial location, arbitrary initial location, different population sizes, and the different number of targets were implemented. It was demonstrated by numerous experiments that SRPB had excellent stability, quick source seeking, a high number of located sources, and a low collision rate in various search strategies.

## 1. Introduction

Steering a group of autonomous robots to search the targets is a well-studied problem due to its numerous important applications. There are many significant applications for target search, including searching and rescuing in a hazard environment [1], environmental monitoring [2], perception in battlefield [3], locating gas leakage, odor source detection, etc. In these scenarios, the robots can sense the environment, collecting and exchanging measurements of the targets, and to exploit this information to guide their movements. The goal is to search the targets in the minimum time while avoiding collisions with other robots.

There are many algorithms to complete the task of a multi-target search. According to the richness of target information, the algorithms of the target search can be divided into three categories. The first is information-lack. In this category, the environment is much larger than the range of communication and sensing, and there is no information about the targets. The goal is to maximize environmental coverage while minimizing overlaps. Search pattern, random walk, search map, digital pheromone [4], Glasius bio-inspired neural network (GBNN) [5,6], and optimization algorithms are the typical algorithms. The search pattern, such as zigzag and spiral [7], can effectively cover a given domain with fewer area overlaps, but it lacks the flexibility to work in a changeable environment. GBNN and digital pheromone are similar to the search map. The search map can improve flexibility and efficiency, but this algorithm is hard to implement in practical scenarios because too much information is exchanged in direct communication that communication devices cannot afford, and there is no suitable way to implement indirect communication [8]. The random walk is the most flexible strategy, but random actions for exploration are inefficient. There are many random strategies, and they are suitable for different situations. For example, Lévy flight [9] is suited to the situation that the environment is large, and the distribution of targets is sparse. Brownian motion is efficient when the targets are abundant, and the environment is small [10]. The optimization algorithms are usually applied to optimization problems [11,12]. Some problems, such as routing problems [13], controlling problems [14], can be converted into optimization problems. The problem of target search can also be converted into an optimization problem, and it can be solved by optimization algorithms. Particle swarm optimization, genetic algorithm are used to solve this problem in [15], but this way is offline, and it has central coordination. The movement of robots is controlled by central coordination. It is time-consuming and lacks flexibility.

The second is information-rich. In this case, the range of communication and sensing are larger or slightly smaller than the environment, and robots in the environment can communicate with each other. There is rich information about the targets, and the goal is to seek the sources quickly. For this task, the methods can be divided into two categories [16]: behavior-based methods and automatic methods. Behavior-based methods, including finite state machine (FSM), swarm intelligence, and formation, are the most concerned methods. Formation methods, including triangular formation [17], rectangular formation [18], and circular formation [19], can estimate the gradient of source, but these methods need to maintain a predefined formation, and they lack flexibility. In the FSM methods, the robot behaviors are abstracted as states and response conditions are used to switch states. Jie Li etc. [20] proposed probabilistic finite state machine ( PFSM). It applies the random walk strategies to the information-lack area, and the triangle formation has been introduced for the information-rich area. The essence of FSM is a combination of different methods. Swarm intelligence is the most common behavior-based method. The motivation of swarm intelligence applying in source seeking is to imitate the foraging behavior of biology groups. Different from optimization algorithms, swarm intelligence regards a solution generated in optimization algorithms as a robot, and the fitness value is the signal strength measured at the robot’s position. It is an online method, and the robot determines the next movement by itself. Robots can sense the environment, collecting and exchanging measurements of targets, and to exploit this information to guide their movements. Ant colony optimization (ACO) [21], particle swarm optimization (PSO) [22,23,24] are widely used for source seeking. Zou R etc. [25] established the equivalence between the particles generated in the PSO algorithm and the seeking robots in the group, and simple collision-avoidance tactics were introduced to realize the cooperative source seeking.

The third is information-weak. Most signals are weak because of fast decay sources, multiple sources, and a large environment. The sources fast decay at a certain distance. Beyond the distance, no signal can be measured. Besides, sometimes there are multiple sources in an environment. The goal is to find sources as many as possible in a certain time to search the sources quickly with weak information and to allocate the robots between different sources autonomously. Relevant research focuses on swarm intelligence because animals can complete this task with limited sensing, limited communication, and local interaction [26]. The typical algorithms are particle swarm optimization (PSO), firefly algorithm (FA), glowworm swarm optimization (GSO), bee swarm optimization (BSO), and firework algorithm [27]. FA and GSO are similar. The robot in the FA is influenced by all of the neighbors that are superior to its own. The robot in the GSO selects a neighbor that has a fitness value higher than its own and moves toward it. Besides, when there is no signal strength emitted by the sources, FA can explore the area, but GSO doesn’t. In [28], based on FA, the levy flight was introduced, and multiple sources seeking was achieved, but this method is inefficient because of the many overlaps generated by levy flight. In [29], based on GSO, the practical robots were used to seek the light source in an information-rich environment. In [30], the authors proposed the multiple extrema seeking algorithm (MESA). At first, robots find neighbors and construct groups. Once forming a group, robots will complete the task of target search based on GSO. Although robots in this strategy can seek multiple sources, the number of targets is known. Besides, this method loses the distribution information of the initial robots’ location. Bee swarm optimization [31] has defined the scout, onlooker, and experienced forager, and different characters undertake different tasks. Particle swarm optimization has also been applied in multiple sources seeking [32], and some variations of PSO, such as robotic particle swarm optimization (RPSO) [33], adaptive-RPSO (A-RPSO) [34], have been introduced, but the scene is simple.

Although there have been some achievements about the information-weak or multiple sources seeking, there are many realistic constraints in a practical environment, and many scenarios, such as maritime rescue, are to seek multiple weak sources. Firstly, limited perception limits the acquisition of signals. Since the distance to the targets or obstacles is too far, and the capability of the sensor is limited, the robots cannot get enough information. Besides, the sources’ fast decay in a certain distance also limits the perception of robots. Secondly, limited communication influences cooperation among robots. Exchanging information is essential for cooperation among robots. If there is no information exchange among robots, the cooperation will not happen. Thirdly, a finite working time of robots influences the number of located sources. In reality, autonomous robots are always constrained by fuel consumption. Fourthly, an unknown number of targets makes it impossible to adopt a proper number of robots, and it requires the robots to keep exploring the environment and autonomously assign the task among sources. Fifthly, unknown extrema make it hard for robots to determine when to stop searching. In real life, the initial location of robots is arbitrary. It requires that the seeking strategy should be stable and universal. Finally, since robots are usually autonomous, and they are limited by the range of communication, there is no central coordination to instruct the motion of robots. It requires that the cooperative strategy is distributed and online.

Considering the above constraints, including a limited range of communication and sensing, a limited working time, unknown sources, unknown extrema, the arbitrary initial location of robots, non-oriented search, and no central coordination, a novel cooperative strategy is proposed. It is inspired by pedestrian behavior in subway/railway stations. The robotic behavior is described as four rules, including (a) Robot can exploit the information about sources and environment. (b) The movement of the robot is influenced by other robots. (c) Robots are attracted by other robots. (d) A large group of robots divides into small groups. Several experiments show that swarm robotic pedestrian behavior (SRPB) has excellent stability, quick source seeking, a high number of located sources, and a low collision rate.

The contributions of this paper were as follows. (a) Inspired by pedestrian behavior and considering many realistic constraints, swarm robotic pedestrian behavior (SRPB) was proposed. (b) The performances of different cooperative strategies were evaluated in terms of average time to find the first, the half, and the last source, the number of located sources and the collision rate. Numerous experiments with different signals, fixed initial position, arbitrary initial location, different population sizes, and a different number of targets were implemented. Compared with various search strategies, including PSO, RPSO, A-RPSO, GSO, FA, and Levy flight search (LFS), in several experiments, SRPB had excellent stability, quick source seeking, a high number of located sources, and a low collision rate.

The rest of the paper is organized as follows. In Section 2, two categories of the searching algorithms are introduced. In Section 3, the problem description of multiple weak sources seeking and some assumptions are stated. In Section 4, the proposed strategy is described in detail. In Section 5, experimental results and discussions are presented. Finally, the work is concluded in Section 6.

## 2. Related Work

There are two categories of multiple sources seeking algorithms. One is swarm intelligence algorithms, while the other is random walk strategies. Swarm algorithms focus on seeking sources in an information-rich environment. Random walk strategies are applied in an information-lack environment. When the number of sources is small, and the distribution of targets is sparse, both information-rich area and information-lack area exist. Meanwhile, when the source sites are abundant, there is only the information-rich area. In reality, since the number of sources is unknown to robots, there are not only the information-rich area but the information-lack area.

### 2.1. Swarm Intelligence Algorithms for Source Seeking

Seeking multiple sources in an unknown environment is difficult for autonomous robots because there are many constraints, including a limited range of communication and sensing, a limited working time, unknown sources, unknown extrema, arbitrary initial location of robots, non-oriented search, and no central coordination. Swarm intelligence, such as PSO, GSO, and BSO, gives a natural idea to complete the searching task. In the swarm intelligence, a robot is regarded as a solution, and the signal strength measured at the robot’s location is taken as the fitness. The cooperation of robots is the solution procedure of the swarm intelligence algorithms. In this part, five strategies inspired by swarm intelligence are introduced as follows:

**Multiple targets PSO:** In [32], particle swarm optimization was used in the multi-target search. The author proposed a dynamically weighted value *w_RSS_*. *w_RSS_* decreases when the distance to the target becomes large and increases when the robot approaches the target. In Equation (1), *w* is the inertia weight. *c*_1_, *c*_2_ are the acceleration constants. *pbest_i_* is the position with the best fitness value for robot *i* in the process. *lbest_i_(t)* is the position with the best robot in the local swarm at time *t*. *r*_1_, *r*_2_ are uniformly distributed random numbers within (0, 1). $x_{i}(t)$ is the position of robots *i* at time *t*, and $v_{i}(t)$ represents the velocity of robot *i* at time *t*.

(1) $$v_{i}(t+1)=(w\cdot v_{i}(t)+c_{1}r_{1}(pbest_{i}-x_{i}(t))+c_{2}r_{2}(lbest_{i}(t)-x_{i}(t)))\cdot w_{RSS}$$

**RPSO:** Robotic particle swarm optimization [33] is an extension of PSO. The obstacle avoidance has been introduced to the velocity. In Equation (2), *c*_3_ is the collision coefficient, *r*_3_ is a random number. In this paper, we regarded the *gbest* as the location of robots with the best fitness in the local swarm. In this paper, each robot considered the other robots within its sensing range as moving obstacles. The equation of velocity is shown in (3).

(2) $$v_{i}(t+1)=w\cdot v_{i}(t)+c_{1}r_{1}(pbest_{i}-x_{i}(t))+c_{2}r_{2}(gbest-x_{i}(t))+c_{3}r_{3}(p_{i}(t)-x_{i}(t))$$

(3) $$v_{i}(t+1)=w\cdot v_{i}(t)+c_{1}r_{1}(pbest_{i}-x_{i}(t))+c_{2}r_{2}(lbest_{i}(t)-x_{i}(t))+c_{3}r_{3}(p_{i}(t)-x_{i}(t))$$

**A-RPSO:** Adaptive robotic PSO [34]. The velocity equation of A-RPSO, shown in Equation (4), is the same with the velocity equation of RPSO, except the inertia weight *w_i_*(*t*). The evolutionary speed factor $h_{i}^{t}$ and aggregation degree *s* are introduced to adjust the inertia weight and other parameters. These factors help to keep diversity between robots. Meanwhile, *c*_2_ and *c*_3_ are influenced by the group size and the change of fitness. Each robot in the A-RPSO considers the other robots within its sensing range as moving obstacles.

(4) $$v_{i}(t+1)=w_{i}(t)\cdot v_{i}(t)+c_{1}r_{1}(pbest_{i}-x_{i}(t))+c_{2}r_{2}(gbest-x_{i}(t))+c_{3}r_{3}(p_{i}(t)-x_{i}(t))$$

**GSO:** Glowworm swarm optimization [35]. A glowworm considers other glowworms that are located within its decision radius and those with higher luciferin value than its own as neighbors, and the glowworm selects a neighbor using a probabilistic mechanism and moves to it. There are three stages to update the position in the GSO. At first, the luciferin update rule is given by Equation (5). *ρ* is the luciferin decay constant (0 < *ρ* < 1). *γ* is the luciferin enhancement constant. *l_i_*(*t*) represents the luciferin level associated with glowworm *i* at time *t*. *J*(*x_i_*(*t*)) is the signal strength taken at glowworm *i* ‘s location at time *t*. Each glowworm selects, using a probabilistic mechanism, a neighbor that has a higher luciferin than its own and moves toward it. The probability of moving to a neighbor j is presented by Equation (6). U*_i_*(*t*) = {*J*:*d_ij_*(*t*) < $r_{d}^{i}(t)$; *l_i_*(*t*) < *l_j_*(*t*)} is the set of neighbors of glowworm *i* at time *t*. $r_{d}^{i}(t)$ is the decision radius of glowworm *i* at time *t*. The update equation of position is shown in (7). Finally, the decision radius is updated by (8). *n_t_* is the maximum size of a group. *N_i_*(*t*) is the number of neighbors in U*_i_*(*t*). In this paper, a random component was introduced to the GSO to help robots to explore the area when there is no neighbor.

(5) $$l_{i}(t+1)=(1-\rho )l_{i}(t)+\gamma J(x_{i}(t))$$

(6) $$p_{ij}(t)=\frac{l_{j}(t)-l_{i}(t)}{\sum_{k\in \;U_{i}(t)}l_{k}(t)-l_{i}(t)}$$

(7) $$x_{i}(t+1)=x_{i}(t)+s\cdot (\frac{x_{j}(t)-x_{i}(t)}{\left\|x_{j}(t)-x_{i}(t)\right\|})$$

(8) $$r_{d}^{i}(t+1)=\min\{r_{s},\max\{0,r_{d}^{i}(t)+\beta (n_{t}-N_{i}(t)\}\}$$

**FA:** Firefly algorithms [36]. It is similar to GSO. The robot in the FA is influenced by all of the neighbors that are superior to its own. The attractiveness is proportional to fitness, and it decreases as the distance between robots increases. If no one is higher than its own, the robot will move randomly. The movement that a firefly *i* is attracted by firefly *j* is described as:

(9) $$x_{i}(t+1)=x_{i}(t)+\beta_{0}e^{-\gamma r_{ij}^{2}}(x_{j}-x_{i})+\alpha \cdot \xi_{i}$$

where $\xi_{i}$ is a random vector. Paper [28] shows that levy flight distribution is more effective than the Gaussian distribution in global searching. So, in this paper, $\xi_{i}$ was a levy flight random vector.

### 2.2. Random Walk Strategies

In an information-lack environment, random walk is the most flexible strategy. Brownian motion and Levy flight search are commonly used. Brownian motion is efficient when the area is small, or the number of robots is large. Levy flight search is used when the distribution of the targets is sparse, and the area is large. Since the width and length of the environment are larger than the maximum speed, the Levy flight search is used to compare with other algorithms.

LFS: Levy flight search [9]. In this model, the speed vector of robots obeys the power-law distribution, and it can get from Equation (10). Where *a* ~ *N*(0, *σ*^2^), *b* ~ *N*(0, 1) are two independent random variables that have a normal Gaussian distribution.

(10) $$v=\frac{a}{{\left|b\right|}^{\frac{1}{\alpha }}}$$

## 3. Problem Description

Consider *N_t_* (*N_t_* ≥ 1) sources distributed in a *W* × *L* environment. These sources can emit some kind of measurable signals, and they could be the electromagnetic signal, the light signal [37], the thermal signal, the acoustic signal [38], even the odor signal [39], and so on. The positions of sources, the spatial distribution of the signal field in the space, and the number of sources are unknown to robots, but each robot can measure the strength of the signal emitted by the sources. Besides, there is the maximum strength at the location of sources, and robots can measure the signal strength at the robots’ position [40]. The goal is that a group of robots consisted of *N_r_* (*N_r_* >> *N_t_*) autonomous robots seek the sources simultaneously [41]. In this paper, there have been some assumptions about this problem.

**Assumption 1:** the boundary of the environment is known. There are *N_t_* (*N_t_*≥1) sources distributed randomly in the environment. $Q=\{q_{1},q_{2}\ldots \;,q_{n}\},q_{i}\in {\mathbb{R}}^{n\times 2}$ is the set of position vectors of sources. Different sources are represented as $\tau_{Q}=\{1,2,\ldots ,N_{t}\}$. Besides, the distance between two adjacent sources is more than 2*R_c_*, where *R_c_* is the communication range of robots. It can be described as:

(11) $$\min\left\|q_{i}-q_{j}\right\|>2R_{c},\forall i,j\in \tau_{Q},i!=j$$

In this paper, there were no other methods to distinguish different sources except the received signal strength. Combining the maximum strengths of signal and position of sources could help robots distinguish different sources. If the maximum strengths of sources are known, different sources can be recognized by the maximum strengths and different locations. In reality, the extrema of sources are unknown. For example, the power of sources is unknown in the sea rescue and battlefield awareness. Therefore, robots can only distinguish different sources by the signal strength taken at robots’ positions and the information of neighborhood robots. Since the robot in swarm intelligence is attracted by neighbors who have high strengths, the source with low power is ignored when the distance between two sources is too close. If the maximum signal strength between the two sources is the same, robots will oscillate between two sources. The communication range of robots is always regarded as the attractive range in multi-target search. If the distances between the two sources are greater than 2*R_c_*, it will avoid the above situations, and robots can seek different sources simultaneously.

**Assumption 2:** in this paper, two signal distributions were considered. One is the isotropic signal (12), and the other is the anisotropic signal (13).

(12) $$l_{x,y}^{i}=e^{-\frac{2\left\|q_{i}-p_{x,y}\right\|}{R}},i\in \tau_{Q}$$

where *R* is the effective range of radiation, $l_{x,y}^{i}$ is the signal strength of source *i* at position (*x*, *y*).

(13) $$l_{x,y}^{i}=e^{-r_{i}^{T}S_{1}^{i}r_{i}}+e^{-r_{i}^{T}\Theta_{\pi /4}^{T}S_{2}^{i}\Theta_{\pi /4}r_{i}},i\in \tau_{Q}$$

$\Theta_{\pi /4}$ represents the π/4 rotation matrix, and $r_{i}$ is the position vector. The signal distribution in an environment can be represented by:

(14) $$l_{x,y}=\sum_{i\in \tau_{Q}}l_{x,y}^{i}$$

**Figure 1:**
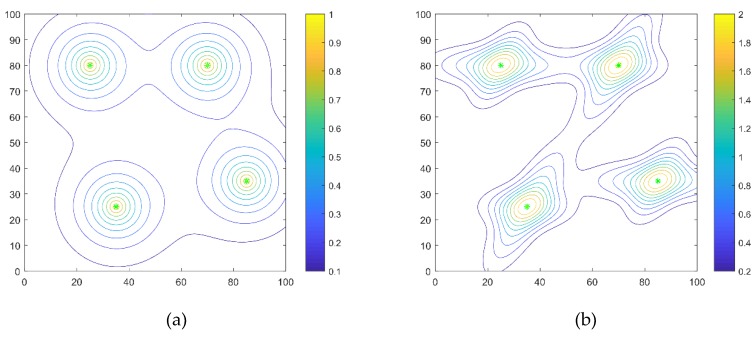
The received signal strength distribution. (**a**) the received signal strength distribution of isotropic signals; (**b**) the received signal strength distribution of anisotropic signals.

Figure 1a,b shows the distribution of four sources in 100 m × 100 m. The locations of the four sources are at position *q*_1_(35,25), *q*_2_(25,80), *q*_3_(70,80), *q*_4_(85,35), respectively. In Figure 1a, *R* is equal to 10 m. In Figure 1b, $S_{1}^{1}=S_{1}^{3}=\frac{1}{50}\left[\begin{array}{cc} 0.23 & 0\\ 0 & 1 \end{array}\right]$, $S_{1}^{2}=S_{1}^{4}=\frac{1}{50}\left[\begin{array}{cc} 0.09 & 0\\ 0 & 1 \end{array}\right]$, $S_{2}^{1}=S_{2}^{3}=\frac{1}{50}\left[\begin{array}{cc} 1 & 0\\ 0 & 0.09 \end{array}\right]$, $S_{2}^{2}=S_{2}^{4}=\frac{1}{50}\left[\begin{array}{cc} 1 & 0\\ 0 & 0.23 \end{array}\right]$.

**Assumption 3:** there are *N_r_* (*N_r_*≫*N_t_*) robots. It can be represented as $\tau_{R}=\{1,2,\ldots ,N_{r}\}$. The position of robots are $P=\{p_{1},p_{2},\ldots ,p_{m}\},p_{j}\in {\mathbb{R}}^{m\times 2}$.

**Assumption 4:** that the source *k* is located by the robot *i* can be defined as:

$$\left\{\;\exists i\in \tau_{R},\forall k\in \tau_{Q}\;|\;\left\|q_{k}-p_{i}\right\|\leq r_{s}\;\right\}$$

**Assumption 5:** the communication and sensing ranges are smaller than the environment, and there is no method to far communication. It can be represented as $W,L\gg R_{c}>R_{s}>r_{s}$. *W* and *L* are the width and length of the environment, respectively.$R_{c}$ is the radius of communication.$R_{s}$ is the radius of sensing. In reality, there are many obstacles, including static, dynamic obstacles. In this paper, each robot considered the other robots within its sensing range as moving obstacles. The repulsive effect in Section 4 works in the sensing range. When the sources are within the range of radius $r_{s}$ of robots, the sources are defined as “located”.

Under these assumptions, the problem is to design a strategy that robots can locate multiple sources and autonomous construct groups. The goal is to seek the sources in the minimum time while avoiding collisions with other robots.

## 4. Proposed Algorithm

Pedestrian behavior is self-organized behavior. It supports an efficient motion in subway/railway stations. Pedestrians exhibit different behavior in the same environment at the same time: they will try to reach the desired destination, and they keep a limited distance from the other pedestrians, also propelled toward their destination by the other pedestrians. Sometimes a large group of pedestrians divides into small groups [42]. The pedestrian behavior is used in distributed autonomous robotic systems because there are some similarities between pedestrians and swarm robots. Firstly, swarm robots and pedestrians decide the next movement with limited observation and calculation. Secondly, the behavior of pedestrians in subway/railway stations is similar to robot navigation [43] and target search. For example, in a search scenario, swarm robots don’t know the specific location of the destination, but they need to reach the destination with limited information. Thirdly, the acquired information, including limited vision information, partial information of targets, is similar. Therefore, the pedestrian behavior will provide a great way to solve the problem of multi-target search.

There are many models to depict pedestrian behavior, such as decision-field-theory [44], social force model [45]. The social force model is similar to swarm intelligence algorithms. It introduces several forces to describe the effects of pedestrian behavior. Based on the social force model, swarm robotic pedestrian behavior (SRPB) is presented. In this paper, there were four rules that determined the movement of robots. (a) The robot can exploit information about sources and the environment. (b) The movement of the robot is influenced by other robots. (c) Robots are attracted by other robots. (d) A large group of robots divides into small groups. In the following, the main effects of swarm robotic pedestrian behavior are introduced in detail.

### 4.1. Main Effects of Swarm Robotic Pedestrian Behavior

1. The robot can exploit the information about sources and environment.

In multi-source seeking, little information about sources and environment can be used, but there are two classes of information to exploit. The first is the environmental size. When there is no signal strength at robots’ position or robot is alone, the boundary of the environment can help robots to visit the given area. The virtual match points are introduced to help robots explore the environment. The number of virtual match points is calculated by:

(15) $$N_{m}=round(\frac{W}{2R_{c}})\cdot round(\frac{L}{2R_{c}})$$

where *W, L* are the width and length of the environment, respectively. $R_{c}$ is the range of communication. $N_{m}$ is the number of virtual match points. *round()* is the round operator. The set of virtual match points is represented by:

(16) $$U_{V}(i)=[R_{c}+2(i-1)R_{c},R_{c}+2(i-1)R_{c}],i\in \{1,2,\ldots ,N_{m}\}$$

Each robot has a set of virtual match points. When the robot is alone, the position of attractive effect is the nearest point in the virtual match point set of this robot. If the robot reaches this point without finding a neighbor, the point will be excluded, and the robot chooses the next virtual match point and moves to it. Virtual match points can avoid revisiting the already covered area. Besides, when the repulsion radius is larger than the decision radius, the virtual match points where the distance to the robot is less than $2R_{c}$ are excluded to avoid revisiting the already located sources.

The second class of information is the already visited position and the corresponding strength. When there is no neighbor to cooperatively estimate the gradient of the source, the individual history effect is introduced to help robots move toward the strong signal area. The individual history effect is defined as:

(17) $$e_{h}^{i}(t)=\frac{p_{i}^{pbest}(t)-p_{i}(t)}{\left\|p_{i}^{pbest}(t)-p_{i}(t)\right\|}$$

$p_{i}^{pbest}(t)$ is the position with the best fitness value for robot *i* up to time *t* in the process. It provides a little gradient information of the target when the group is small. The individual history coefficient is expressed as (19).

(18) $$h_{i}(t)=\gamma_{0}\cdot e^{-\frac{l^{i}(t)-l_{\min}^{i}+\varepsilon }{l_{\max}^{i}-l_{\min}^{i}+\varepsilon }}$$

(19) $$K_{h}^{i}(t)=h_{i}(t)\cdot e^{-\frac{\left(N_{d}^{i}(t)-1\right)}{2}}$$

$h_{i}(t)$ is the cognitive coefficient of robot *i* at time *t*. $K_{h}^{i}(t)$ is the individual history coefficient of robot *i* at time *t*. $l^{i}(t)$ is the fitness of robot *i* at time *t*. $l_{\min}^{i}$ is the minimum fitness of robot *i* in the motion. $l_{\max}^{i}$ is the maximum fitness of robot *i* in the motion. $\gamma_{0}$ is the maximum cognitive coefficient. It balances local searching and global searching. $N_{d}^{i}(t)$ represents the number of robots within the robot *i*’s decision radius at time *t*. The robots within the robot *i*’s decision radius are defined as $\psi_{d}^{i}(t)=\{j:d_{ij}(t)<R_{d}^{i}(t);j\in \tau_{R}\}$, and $d_{ij}(t)$ is the distance between robot *i* and robot *j* at time *t*. ε is dimensionless.

When there is no signal strength, robots visit the given area with the virtual match points. The virtual match points help the robot avoid revisiting the already covered area. By the way, the update of the virtual match point set is distributed and independent. If one robot has visited an area, the other robots still can visit this area. When the robot is alone, the robot explores the given area with the virtual match points, and it approaches the strong strength area with the individual history effect. Besides, the individual history effect can help robots construct a group quickly because all robots will move towards a strong strength area.

2. The movement of robot is influenced by other robots.

Different from the swarm intelligence algorithm, pedestrian behavior focuses on the repulsive force. In subway/railway stations, pedestrian keeps a safe distance from other pedestrians when the crowd flow is small. When the pedestrian flow is big, the pedestrian is propelled forward. It can be described by the repulsive effect. The robot keeps a safe distance from the other robots, and it can be propelled towards the destination. The repulsive effect is the combination of all repulsive forces. The repulsive force is described as follows:

(20) $$K_{c}^{ij}(t)=\frac{d_{ij}(t)-R_{r}^{i}(t)}{d_{ij}(t)+1},i\in \tau_{R},j\in \psi_{r}^{i}(t)\{k:0<d_{ik}(t)\;\leq R_{r}^{i}(t),k\in \tau_{R}\}$$

$K_{c}^{ij}(t)$ is the influence coefficient between robots *i* and robot *j* at time *t*. $d_{ij}(t)$ is the distance between robot j and robot *i* at time *t*. $\psi_{r}^{i}(t)$ is the robots within the repulsion range of robot *i* at time *t*. $R_{r}^{i}(t)$ represents the repulsion radius of robot *i* at time *t*. The robots within the repulsion range influence the behavior of the focal robot. The closer a robot gets to the focal robot, the bigger the absolute value of the influence coefficient is. The repulsive effect is the sum of all repulsive forces. It can be described by Equation (22):

(21) $$r_{i}(t)=\sum_{j\in \psi_{r}^{i}(t)}K_{c}^{ij}(t)\cdot \frac{p_{j}(t)-p_{i}(t)}{\left\|p_{j}(t)-p_{i}(t)\right\|}$$

(22) $$e_{r}^{i}(t)=\frac{r_{i}(t)}{\left\|r_{i}(t)\right\|}$$

$e_{r}^{i}(t)$ is the repulsive effect. $r_{i}(t)$ is the weighted sum of all repulsive forces. $p_{j}(t)$ is the position of robot *j* at time *t*. In traditional swarm intelligence algorithms, avoiding collisions can only keep a safe distance between robots. In this paper, the repulsive effect could help the robot keep a certain distance from other robots, and it propelled the robot towards the destination. Keeping a safe distance from other robots or propelling the robot to the destination depends on the priority of the focal robot in a group. When robots construct a group, robots in the group calculate their priority coefficients. The maximum value of the priority coefficient of a robot means that there are no neighbors. The minimum value means that there are many neighbors. In this paper, a robot considered other robots that were located within its decision radius and those with higher fitness value than its own as neighbors. The repulsive effect and repulsion radius will elastically change according to the priority coefficient. If the priority coefficient is large, the repulsive effect and repulsion radius will become big, and the swarm will propel this robot to the source, and the attractive effect will become small. If the priority coefficient is small, the attractive effect will become large, and the repulsive effect will become small. In this method, the priority coefficient is the criterion of change of the repulsion radius. It can improve the efficiency of source seeking and avoid collisions between robots. The priority coefficient is calculated by:

(23) $$K_{r}^{i}(t)=\rho \cdot e^{-\frac{\left(l_{gbest}(t)-l^{\;i}(t)+\varepsilon \right)}{\left(l_{gbest}(t)-l_{gworst}(t)+\varepsilon \right)}}$$

ρ is the maximum priority coefficient. It helps robots keep a safe distance between robots and propels robots forward. $K_{r}^{i}(t)$ is the collision coefficient of robot *i* at time *t*. $l_{gbest}(t)$ is the best fitness within the local group. $l_{gworst}(t)$ is the worst fitness within the local group, and $l^{i}(t)$ is the fitness of robot *i* at time *t.* ε is dimensionless. Finally, the repulsion radius will update as follow:

(24) $$R_{r}^{i}(t)=\min(R_{r}^{\max},max(R_{r}^{\min},K_{r}^{i}(t)\cdot R_{r}^{\max}))$$

where $R_{r}^{\max}$, $R_{r}^{\min}$ are the maximum repulsion radius and the minimum repulsion radius, respectively. 

3. Robots are attracted by other robots. 

Pedestrian in an unknown environment tends to follow the person who has a specific objective or has more information about the destination. When a person would like to go to an unknown place, the best way is to follow the people who know this place. In swarm intelligence algorithms, the attractive force determines the convergence of an algorithm. The attractive force is influenced by neighbors, and the decision radius limits the range of neighbors. A robot considers other robots that are located within its decision radius and those with higher fitness value than its own as neighbors, and the robot selects a neighbor using a probabilistic mechanism and moves to it. The set of neighbors can be expressed as $\psi_{n}^{i}(t)=\{j:d_{ij}(t)<R_{d}^{i}(t);l^{i}(t)<l^{j}(t);j\in \tau_{R}\}$. When the robot has higher fitness than robot *i* and the distance to robot *i* is less than $R_{d}^{i}(t)$, the robot is a neighbor of robot *i*. The probability of moving toward a neighbor *j* for robot *i* is given by:

(25) $$pc_{ij}(t)=\frac{l^{j}(t)-l^{i}(t)+\varepsilon }{\sum_{k\in \psi_{d}^{i}(t)}\left(l^{k}(t)-l^{i}(t)\right)+\varepsilon }$$

$pc_{ij}(t)$ is the probability of moving toward a neighbor *j*. $l^{j}(t)$ is the fitness of the neighbor robot *j*. Once one robot *k* is selected, the attractive effect is described as:

(26) $$e_{a}^{i}(t)=\frac{p_{k}(t)-p_{i}(t)}{\left\|p_{k}(t)-p_{i}(t)\right\|},k\in \psi_{n}^{i}(t)$$

The attractive coefficient is influenced by the individual history coefficient and the priority coefficient. It is described as:

(27) $$K_{a}^{i}(t)=(1-K_{r}^{i}(t))(1-K_{h}^{i}(t))$$

When the individual history coefficient is big, the robot is alone, and the attractive effect is small. When an individual history coefficient becomes small, the attractive effect becomes big because the robot joins a group, and it is attracted by other robots. When the priority coefficient becomes big, the repulsive effect becomes big, and the attractive effect becomes small. When the priority coefficient becomes small, the attractive effect becomes large, and the repulsive effect becomes small.

4. A large group of pedestrians divides into small groups.

At the exit of a subway/railway station, pedestrian tends to move towards the exits where there are fewer persons. But in a distributed system, robots can’t recognize the right size of a group because of the limited communication and sensing ranges. Even if the group size is clear, robots can’t decide who will drop out of the current group because the movement of robots is independent. In this part, the self-tuning decision radius is introduced. Combining the decision radius and the repulsion radius can adjust the group size. The decision radius is updated by:

(28) $$R_{d}^{i}(t)=\min(R_{c},max(R_{s},R_{d}^{i}(t)+\beta \cdot (N_{\max}-N_{n}^{i}(t))\cdot e^{-(N_{\max}-N_{n}^{i}(t))}\cdot R_{c}))$$

where $R_{d}^{i}(t)$ is the decision radius of robot *i* at time *t*, and $N_{n}^{i}(t)$ is the number of neighbors of robot *i* at time *t*.$N_{\max}$ is the maximum number of neighbors. The change rate of the decision radius is influenced by β. When the $N_{n}^{i}(t)$ is less than $N_{\max}$, robot *i* is alone or the fitness of robot *i* is the best in the group, so the decision radius will increase. When the $N_{n}^{i}(t)$ is more than $N_{\max}$, robot *i* has the lowest fitness value in the group, so the decision radius decreases sharply. Once the repulsion radius is larger than the decision radius, the virtual match points where the distance to the robot is less than $2R_{c}$ are excluded. Then, the robot *i* will become alone, and the decision radius will increase slowly. In this way, the worst robot will drop out of the group, and the chain effect between robots will help the group to keep a suitable size.

### 4.2. The Equation of Velocity and Position

The equation of velocity is updated by Equation (29), and the equation of position is (30).

(29) $$v_{i}(t+1)=w\cdot v_{i}(t)+K_{h}^{i}(t)\cdot v_{m}\cdot e_{h}^{i}(t)+K_{a}^{i}(t)\cdot v_{m}\cdot e_{a}^{i}(t)+K_{r}^{i}(t)\cdot v_{m}\cdot e_{r}^{i}(t)$$

(30) $$p_{i}(t+1)=p_{i}(t)+v_{i}(t+1)$$

*w* is the inertia coefficient. Inertia provides the reference information for the velocity and smoothes the trajectories of robots.$v_{i}(t)$ is the velocity of robot *i* at time *t*, and it is not more than $v_{m}$. $p_{i}(t)$ is the position of robot *i* at the time *t*. $v_{m}$ is the maximum velocity. 

**Figure 2:**
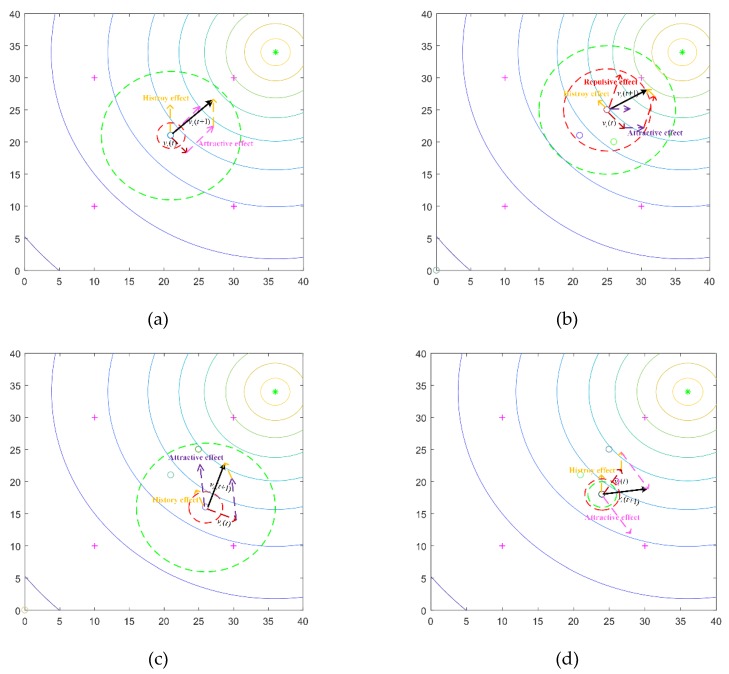
The velocity updating process of a robot in swarm robotic pedestrian behavior (SRPB). (**a**) robot is alone; (**b**) repulsive effect acts as the thrust; (**c**) attractive effect; (**d**) separation.

In Figure 2, we gave some illustrations about the velocity updating process of a robot in SRPB to explain the four rules. The little solid line circles in Figure 2 represent the robots. The red dotted circle is the repulsion radius. The green dotted circle is the decision radius. The purple crosses are the virtual match points. The sources are represented by the green asterisk. Different color arrows indicate different effects. By the way, the attractive effect has two forms.

In Figure 2a,d, the attractive effect is influenced by the virtual match points, and it is also influenced by neighbors, as shown in Figure 2b,c. Different attractive effects are depicted by different colors in Figure 2. In Figure 2a, when the robot is alone, three effects, including inertia, the attractive effect, and the history effect, determine the motion of the robot. The robot selects the nearest point in the virtual match point set as an attractive point. The history effect helps the robot approach the strong signal area. There is no repulsive effect because no robots are within the sensing range of the focal robot, and the velocity is determined by inertia, history effect, and attractive effect. When robots construct a group, the robots calculate the priority coefficient according to the fitness in the group. The repulsion radius is proportional to the priority coefficient. As shown in Figure 2b, the robot is influenced by four effects. Since the robot shown in Figure 2b has the maximum fitness value in the group, the priority coefficient is big, and the repulsion radius becomes large. The attractive effect becomes small. The history effect is small because there are many robots within the sensing range. Other robots in the group propel the robot toward the destination. As shown in Figure 2c, there is no repulsive effect because the repulsion radius is small, and there are no robots within the repulsion radius. The robot is attracted by one of the neighbors. In Figure 2d, the decision radius is tuned by the number of neighbors. When the repulsion radius is larger than the decision radius, the robot drops out of the group and searches other sources. In addition, when robots leave one source, the virtual match points where the distance to the robot is less than $2R_{c}$ are excluded. It can help robots avoid revisiting already located sources. 

### 4.3. The Pseudo-Code of SRPB

In this part, the proposed SRPB algorithm was shown in Algorithm 1. All rules of SRPB were implemented according to the pseudo-code of SRPB.

**Algorithm 1:** The SRPB search strategyinitialize population with random positions and velocitiesgenerate and initialize virtual match point set to each robot do for each time in given time  do for each robot in swarm    Generate decision set $\psi_{d}^{i}(t)$, neighbor set $\psi_{n}^{i}(t)$, repulsion set $\psi_{r}^{i}(t)$
    **If** the robots number of $\psi_{n}^{i}(t)$ is greater than 0      Calculate probabilities of moving to neighbors       Select one robot and update $p_{k}(t)$
    **end**    **If** the robots number of $\psi_{d}^{i}(t)$ is equal to 0      Choose the nearest point in robot’s virtual match point set      update $p_{k}(t)$
    **end**    Calculate repulsive effect according to Equations (20)–(22)    Calculate the history coefficient, the priority coefficient and the attractive coefficient     Update repulsion radius and decision radius    **If** repulsion radius is greater than decision radius      Exclude points where the distance to the robot is less than $2R_{c}$
      Choose the nearest point in the robot’s virtual match point set      update $p_{k}(t)$
    **end**    Calculate the history effect and the attractive effect     Update velocity Equation (29)  **end**
    do for each robot in swarm    Update position Equation (30)    Update robot’s received signal strength at its position     Update $l_{\max}^{i}$ and $l_{\min}^{i}$
    **If** robot reach to the chosen point       Exclude the chosen point    **end**  **end**
**end**


## 5. Simulations and Analysis

In this section, there are several parts to discuss the proposed algorithm. At first, we analyzed the effect of parameters and performed some experiments. Those parameters were taken to complete the experiments of comparison. Secondly, swarm exploration behavior with different signals was shown. Thirdly, several groups of experiments were implemented with different population sizes, different numbers of sources, and different distribution of initial position. The performances of different cooperative strategies, including SRPB, PSO, RPSO, A-RPSO, GSO, FA, and LFS, were evaluated in terms of average time to find the first, the half, and the last source, the number of located sources, and the collision rate. Finally, the analysis of how to implement this strategy, in reality, was given. Besides, different criteria were evaluated by the mean and standard deviation of many experiments, and these were denoted by mI and dI, respectively. mI indicates the searching efficiency of strategies, while dI reflects stability. All experiments were implemented with MATLAB R2017a in windows 10.

Moreover, a collision is defined as: at one moment, the distance between any two robots is less than half of the minimum repulsion radius. The collision rate is equal to the ratio of the collision number to the given time. The average discovery number rate is the ratio of the located sources to the total sources.

### 5.1. Parameter Analysis for the SRPB Strategy

There are four parameters that influence the search efficiency of the SRPB strategy, including *w*, $\gamma_{0}$, ρ, and β. Each parameter was analyzed separately and sequentially, with the other three parameters fixed. The experiments were implemented in a fixed scenario, as shown in Figure 1a, with 20 robots and 4 sources. Firstly, the parameter of the inertia weight *w* was analyzed with $\gamma_{0}=0.48$, ρ=0.8, and β = 0.3. Inertia provides the reference information for the velocity, and it can smooth the trajectories of robots. In most swarm intelligence algorithms, inertia weight is usually within (0, 1), and it needs a large value, so different values within (0.5, 0.98) are used to analyze the algorithm. By the way, before the effect of inertia weight was analyzed, we had already taken other parameters with a proper value. In this paper, $\gamma_{0}$ was to balance local searching and global searching. Exploring sources as many as possible is better for the environment with an unknown number of sources, but the efficiency of source seeking is related to local searching. To balance it, $\gamma_{0}=0.48$ was taken. ρ is used to provide a repulsive force to propel a robot forward and keep a safe distance. It must take a large value. When the repulsive effect acts as a thrust, the attractive effect is small because there are no neighbors, and the attractive effect plays a little role in velocity. So, ρ=0.8 was taken. Finally, β is related to the tuning of the decision radius. In this paper, the self-tuning decision radius was used to determine which robot should drop out of the group. β is the change rate of the decision radius. When the number of neighbors exceeds a value, the decision radius will decrease sharply and then slowly increases. If β is large, the decision radius will increase quickly. It cannot drop out of the group because the robots are attracted by the neighbors within the decision radius. Besides, β cannot be a small value. It makes robots move far away from the neighbor sources because the robot cannot cooperate with other robots. So, β = 0.3 was taken.

**Figure 3:**
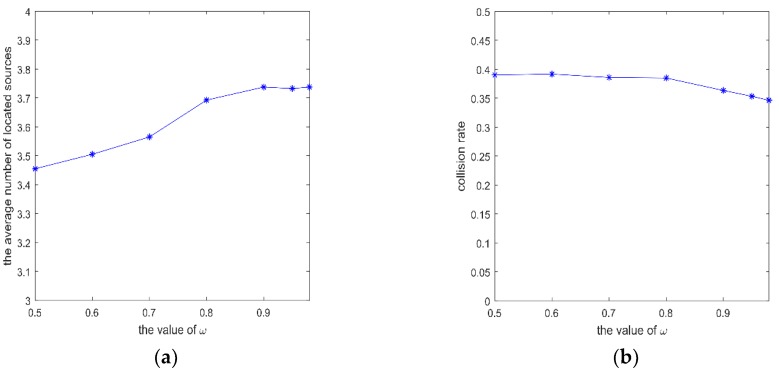

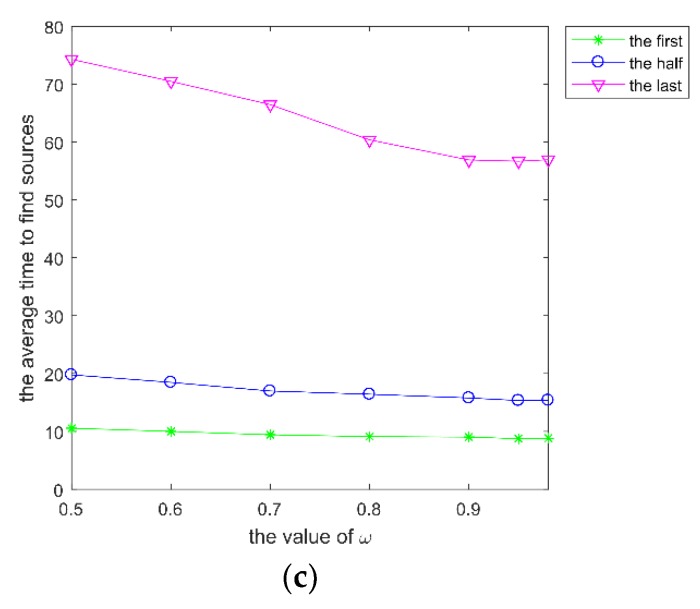
mI of SRPB at different *w*, which varies within (0.5, 0.98). (**a**) the average number of located sources; (**b**) collision rate; (**c**) average time to find different sources.

As can be seen from Figure 3, with the increase of *w*, the average number of located sources increases. The collision rate becomes small, and the average time to located sources decreases. It shows that inertia weight needs to be a large value, and inertia plays an important role in the motion. Inertia provides the reference information for the velocity, especially when there is no information about the environment. Besides, when *w* is greater than 0.9, the collision rate, the average number of located sources, and the average time to located sources remain unchanged. It concludes that the parameter *w* should be big.

**Figure 4:**
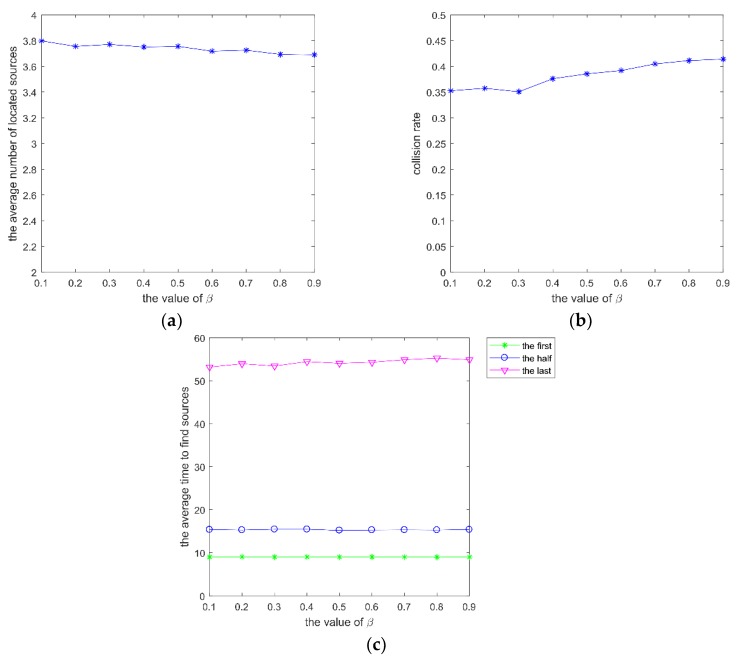
mI of SRPB at different β, which varies within (0.1, 0.9). (**a**) the average number of located sources; (**b**) collision rate; (**c**) average time to find different sources.

Secondly, parameter β, which varies within (0.1, 0.9), was analyzed with $\gamma_{0}=0.48$, ρ=0.8, and *w* = 0.95 in the same scenario. The performance with different β is shown in Figure 4.

The change rate of decision radius is influenced by β. When the number of neighbors exceeds a value, the decision radius decreases sharply. Once the repulsion radius is larger than the decision radius, the robot will become alone and then move to other areas. If β is too big, the robots within the decision radius of the focal robot attract the focal robot all the time, and the group size can’t be adjusted effectively. It can be seen from Figure 4 that the average number of located sources, the collision rate, and the average time to find different sources are poor with the increase of β. 

Thirdly, the parameter $\gamma_{0}$ was analyzed with β = 0.3, ρ=0.8, and *w* = 0.95. $\gamma_{0}$ is to balance local searching and global searching. When the value of $\gamma_{0}$ is greater than 0.5, robots perform global searching first. Local searching is a priority when $\gamma_{0}$ is less than 0.5.

**Figure 5:**
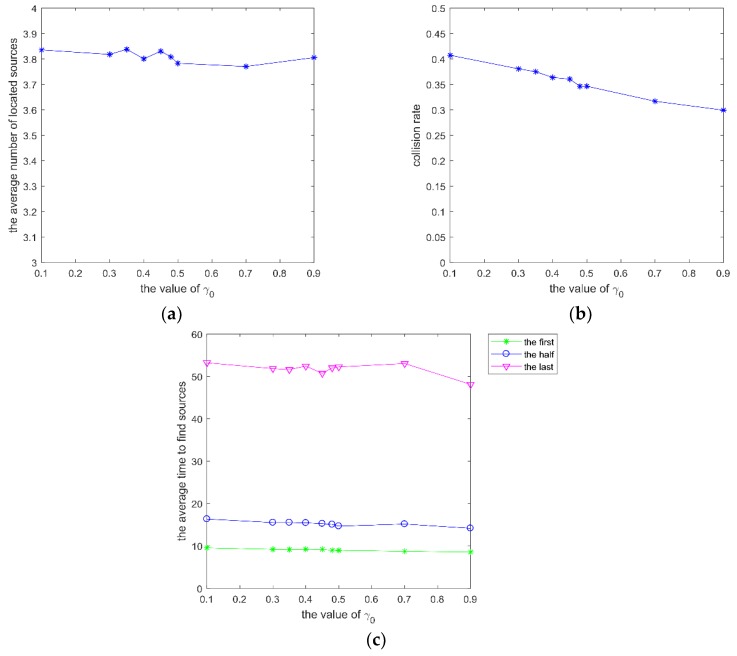
mI of SRPB at different $\gamma_{0}$, which varies within (0.1, 0.9). (**a**) the average number of located sources; (**b**) collision rate; (**c**) average time to find different sources.

Figure 5 shows that a large $\gamma_{0}$ performs well because the history effect can help robots approach the strong signal area. The history effect plays an important role in seeking the source, especially when the robot is alone. Nevertheless, there are two situations that robots become alone. In the initial location, some robots may be alone because of arbitrary location, and they would like to approach the source quickly. It requires a big history effect. When the robot drops out of the group, it tends to explore the other sources. The small history effect helps the robot drop out of the group; otherwise, the robot will always stay at this group. A small history coefficient will help robots divide into several small groups. It also makes robots seek sources as many as possible because the number of located sources is related to the number of groups of robots. In reality, since the number of sources is unknown, $\gamma_{0}$ is smaller than 0.5 to explore more sources.

Finally, parameter ρ was analyzed with β = 0.3, $\gamma_{0}$ = 0.48, and *w* = 0.95. ρ is related to the role of the repulsive effect. 

**Figure 6:**
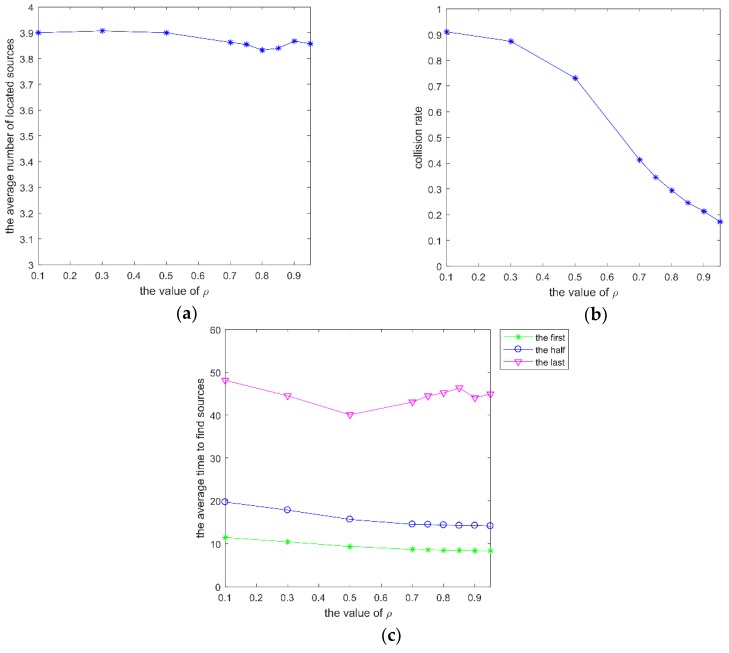
mI of SRPB at different ρ, which varies within (0.1, 0.95). (**a**) the average number of located sources; (**b**) collision rate; (**c**) average time to find different sources.

There are two roles that the repulsive effect plays. One is to keep a safe distance from other robots, and the other is acting as a thrust. As shown in Figure 6b, when the value of ρ is small, robots can’t avoid collisions between robots. When the repulsive effect acts as a thrust, the repulsive effect should be greater than the attractive effect because sometimes the attractive effect and the repulsive effect are contradictory. Therefore, in this paper, ρ was greater than 0.5. When the repulsive effect has a great effect on robots, the attractive effect is small, and the repulsive effect will propel the robot forwards.

### 5.2. Algorithms for Comparison

In this part, all parameters of comparison algorithms are given. Considering fuel consumption, robots can only work in a limited time. It can be determined by the maximum speed and the width and length of the environment. For example, if the environment is 100 m × 100 m, and the maximum speed of robots is 2 m/s, each robot can work for 100 s. In this way, a single robot can’t visit a complete environment. The goal is to minimize the average time to find sources and to maximize the number of located sources. In all experiments, the radius of communication is 10 m. The minimum repulsion radius is 2 m. The maximum repulsion radius is half of the communication radius. The maximum speed is 2 m/s. All algorithms and their corresponding parameter configurations are shown as follows:

PSO: multiple target particle swarm optimization. In [32], multi-target search was considered. Therefore, all parameters are shown as: Inertia weight *w* = 0.9, cognition coefficient *c*_1_ = 1.0, social coefficient *c*_2_ = 1.0.

RPSO: Robotic particle swarm optimization. This method has been used in one target search, and it can be applied in a multi-target search when the *gbest* in the RPSO is regarded as the location of the best robots within the local swarm. In this paper, all parameters were tuned under the same experimental conditions, shown in part 5.1. Inertia weight *w* = 0.95, cognition coefficient *c*_1_ = 1.0, social coefficient *c*_2_ = 2.0, obstacle avoidance coefficient *c*_3_ = 2.0.

A-RPSO: Adaptive robotic particle swarm optimization [33]. Although the experiments of A-RPSO is to seek one source, the A-RPSO is also suited to the multi-target search. All parameters are shown as: Inertia weight *w* = 1, cognition coefficient *c*_1_ = 2.0, *α* = 0.4, *β* = 0.6, *ρ* = 0.4, U = 2, L = 0.5. 

GSO: Glowworm swarm optimization. This algorithm was used for a multi-target search in paper [29]. The parameters are: the luciferin enhancement constant *γ* = 0.6, the maximum size of a group *n_t_* = 4, and *β* = 0.08.

FA: Firefly algorithm. In this paper, all parameters were tuned under the same experimental conditions, shown in part 5.1. Attractiveness *β* = 0.8, light absorption coefficient *γ* = 0.01, random coefficient *α* = 1. 

LFS: Levy flight search. In [20], LFS was applied in a multi-target search, and its parameters are: *α* = 1.5, b = 1.001.

SRPB: Swarm robotic pedestrian behavior. Inertia weight *w* = 0.95, maximum cognitive coefficient $\gamma_{0}=0.48$, maximum priority coefficient ρ=0.8, the change rate of decision radiusβ = 0.3, the maximum number of neighbors $N_{\max}$= 3.

### 5.3. Swarm Exploration Behavior with Different Signals

**Figure 7:**
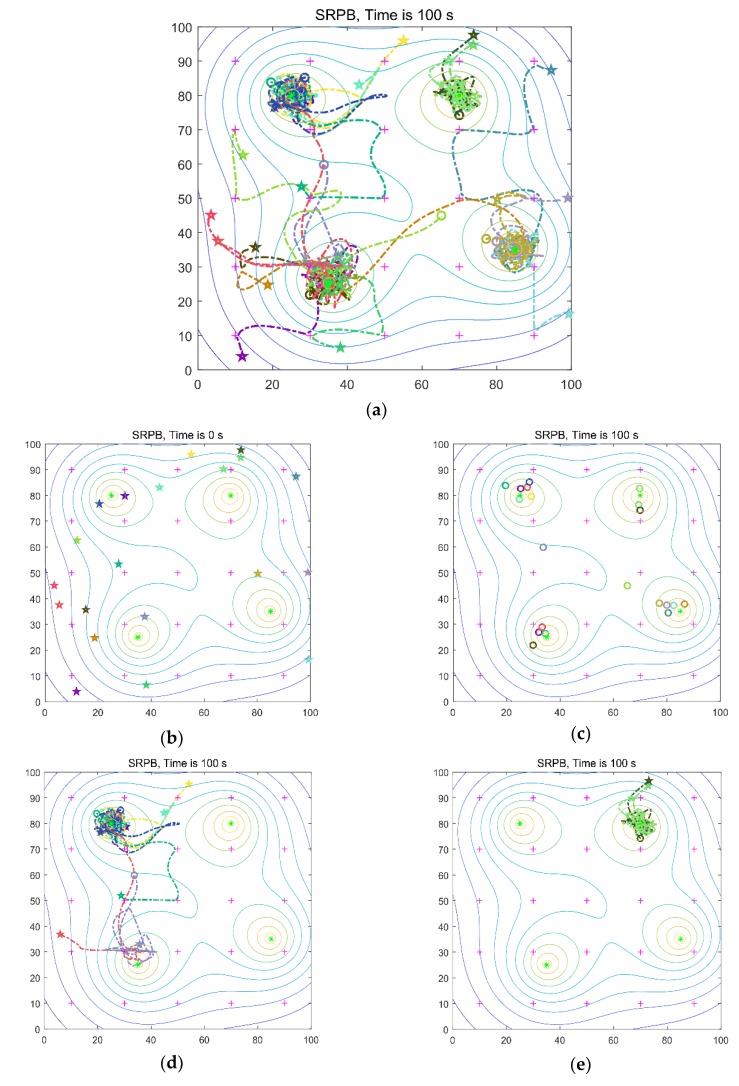

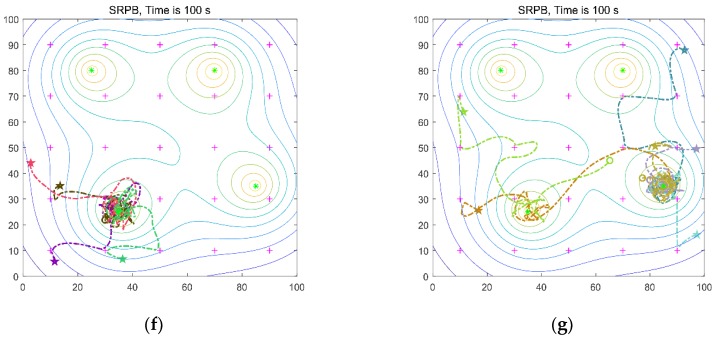
Twenty robots seek four isotropic signal sources. (**a**) robots’ trajectories from initial locations to the extrema; (**b**) initial location of robots; (**c**) final location; (**d**) robots move to the first target; (**e**) robots move to the second target; (**f**) robots move to the third target; (**g**) robots move to the last target.

**Figure 8:**
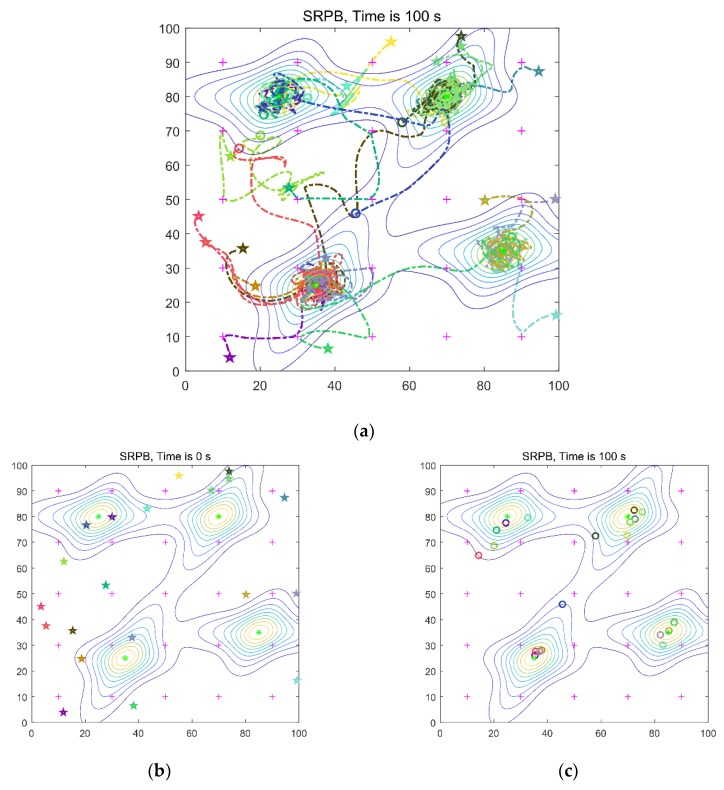

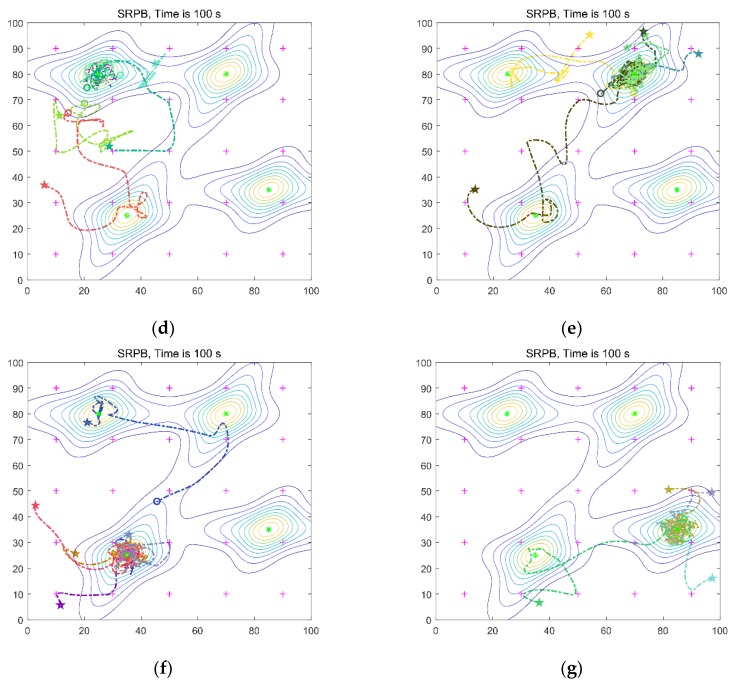
Twenty robots seek four sources of the anisotropic signal. (**a**) robots’ trajectories from initial locations to the extrema; (**b**) initial of robots; (**c**) final location; (**d**) robots move to the first target; (**e**) robots move to the second target; (**f**) robots move to the third target; (**g**) robots move to the last target.

In this part, the swarm exploration behavior with different signals is shown. These experiments implement with four sources and 20 robots in an 100 m × 100 m environment. The four sources are at position q_1_(35,25), q_2_(25,80), q_3_(70,80), q_4_(85,35), respectively. The distribution of the four sources is shown in Figure 1. We gave the robots’ trajectories from initial locations to the extrema, initial arbitrary distribution of the robots, and final location. Besides, the robots’ trajectories from initial locations to different sources are shown, respectively. The limited work time is 100 s, and the initial locations of the robots are arbitrary. In Figure 7 and Figure 8, the purple crosses are virtual match points. The sources are represented by the green asterisk. The little circle represents the final location of a robot. The pentagram represents the initial location of a robot. The dotted line is the robot’s trajectory, and different colors represent different robots.

Firstly, the isotropic signals shown in Figure 1a are used. The time to find the first, the half, and the last sources are 10 s, 23 s, 23 s, respectively. The collision rate is equal to 0.26, and the number of located sources is equal to 4. The robots’ trajectories are shown in Figure 7a.

The anisotropic signals shown in Figure 1b are used. The time to find the first, the half, and the last source are 7 s, 20 s, 26 s, respectively. The collision rate is equal to 0.29, and the number of located sources is equal to 4. The robots’ trajectories are shown in Figure 8a.

### 5.4. Stability between Different Algorithms

In source seeking, different population sizes, different number of sources, and the size of the environment influence the performance of the swarm intelligence algorithms. A different initial position distribution of robots and the random effect of swarm intelligence algorithms also have an impact on the stability of source seeking. Some random parameters in a swarm intelligence algorithm can keep a diversity of solutions, but the algorithm with too many random effects is inefficient and unstable in source seeking. In reality, the stability of source seeking requires that the strategy can work in arbitrary initial locations and seek the targets with approximate numbers in a fixed initial location. In this part, the experiments of source seeking with the fixed initial location and the same sources are implemented in the environment, shown in Figure 1. At first, 20 robots are randomly placed in the environment, and then experiments with the same initial location are implemented 400 times. The mean (mI) and standard deviation (dI) of many experiments in different criteria are used to evaluate the stability of different algorithms.

**Figure 9:**
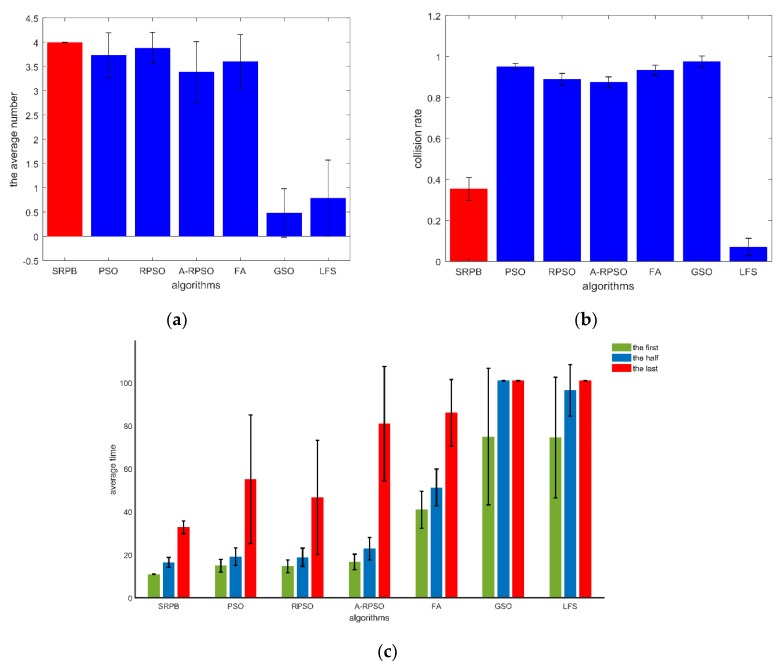
The performance of different strategies with the same initial position of robots in 100 m × 100 m. (**a**) the number of located sources; (**b**) collision rate; (**c**) the time to find sources.

Figure 9 gives the error histograms of different criteria. In Figure 9, an algorithm with a high standard deviation means that the same algorithm in many experiments shows different performances in the same condition. As shown in Figure 9b, the average number of located sources (mI) is approximate in SRPB, PSO, RPSO, A-RPSO, and FA, but the SRPB has a slight advantage than other algorithms. Besides, SRPB has the lowest standard deviation (dI) between all algorithms. PSO, RPSO, A-RPSO, FA, GSO, and LFS have a high standard deviation (dI). It means that these algorithms, including PSO, RPSO, A-RPSO, FA, GSO, and LFS, are unstable. These algorithms are influenced by the random effect. According to the stability and an average number of located sources, shown in Figure 9a, these strategies can be sorted as SRPB>RPSO>PSO≈FA>A-RPSO>LFS>GSO. SRPB is more stable than other algorithms. In Figure 9b, according to the collision rate, SRPB is better than all strategies except the LFS, and it can be sorted as LFS>SRPB>A-RPSO≈RPSO>PSO≈FA>GSO. In Figure 9c, the performance of SRPB, PSO, RPSO, A-RPSO in terms of the time to find the first and the half sources are approximate. SRPB is better than other strategies in terms of the time to find the last source, and it shows great stability. The other strategies have a high standard deviation, so these algorithms can be sorted as SRPB>RPSO>PSO>A-RPSO>FA>GSO>LFS.

In conclusion, SRPB is more stable than other algorithms in the same condition, and it performs great stability and has a better performance than other algorithms.

### 5.5. Different Population Sizes

In this part, experiments with different population sizes and different initial position distribution of robots are implemented in the environment, shown in Figure 1a. Eight tests are carried out with 12, 15, 18, 20, 25, 30, 40, 50 robots, in turn, and the working time of robots is 200 s. Each test is implemented 400 times, and the initial position of robots is updated every time. The performance of SRPB is compared with PSO, RPSO, A-RPSO, GSO, FA, and LFS. By the way, dI is the standard deviation of many experiments.

**Figure 10:**
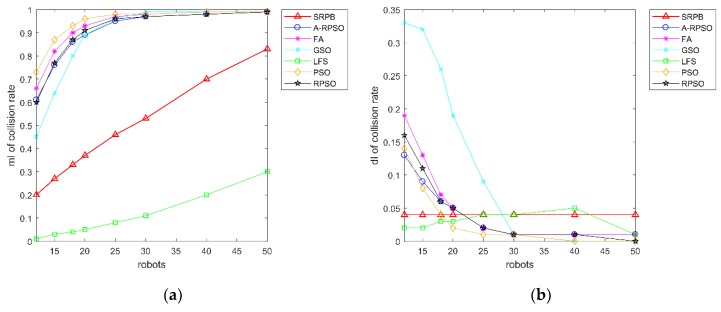
Collision rate of strategies with the different population sizes and the different initial positions of robots. (**a**) mI of collision rate; (**b**) dI of collision rate.

There is a contrast curve of the collision rate of search strategies in Figure 10. Figure 10a shows that the collision rate of different algorithms grows large with the increase of population sizes. When the population size of robots exceeds 20, the collision rate of PSO, RPSO, A-RPSO, FA, and GSO is greater than 80%. SRPB shows an obvious growth, but its collision rate is lower than the other strategies, including PSO, RPSO, A-RPSO, FA, and GSO. The collision rate of LFS is the lowest due to a lack of cooperation. Besides, PSO, RPSO, A-RPSO, FA, GSO have a large standard deviation when the population sizes of robots are less than 30, and the standard deviation of the collision rate of SRPB remains unchanged. Therefore, SRPB is more stable than other strategies. According to the collision rate, these strategies can be sorted as LFS>SRPB>GSO>A-RPSO≈RPSO>FA>PSO.

**Figure 11:**
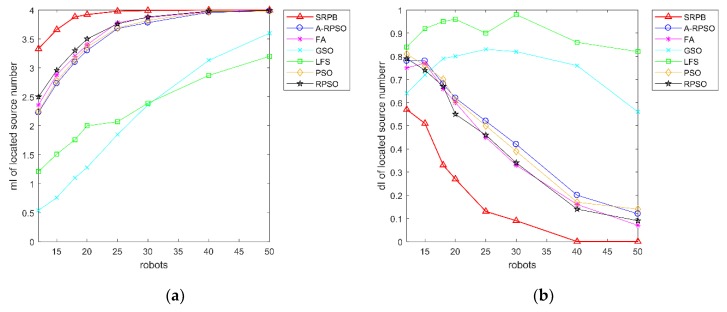
The number of located sources with different population sizes and the different initial positions of robots. (**a**) mI of the number of located sources; (**b**) dI of the number of located sources.

As we can see from Figure 11, SRPB is superior to other algorithms when the population size of robots is lower to 30. When the population size of robots exceeds 30, robots in these algorithms can find the approximate number of sources. Besides, Figure 11b shows that SRPB is slightly influenced by the different initial positions of robots, and it is more stable than other strategies. Hence, it can be sorted as SRPB>RPSO>FA>PSO≈A-RPSO. By the way, LFS is superior to GSO when the population size of robots is less than 30. Once the population size of robots exceeds 30, GSO outperforms LFS because GSO cannot move without neighbors, and it suits to work in the large population size of robots. 

**Figure 12:**
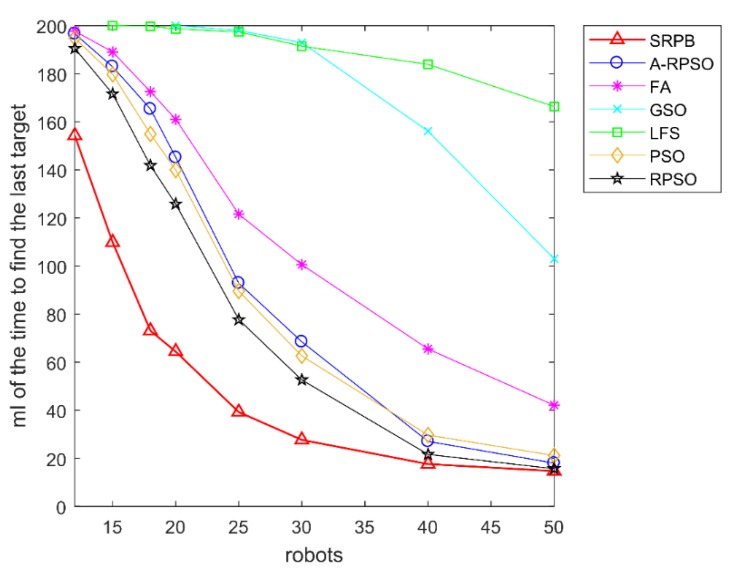
Average time to find the last source in the different population sizes and the different initial positions of robots.

According to the time to find the last source, shown in Figure 12, SRPB is superior to other algorithms, and these algorithms can be sorted as SRPB>RPSO>PSO≈A-RPSO>FA>GSO>LFS.

In conclusion, the proposed algorithm SRPB performs well than other algorithms, and it has excellent stability. For all algorithms, with the number of robots increasing, the time to find the last source decreases, and the number of located sources and the collision rate gradually increases. 

### 5.6. Different Numbers of Targets

In this part, the search efficiency of comparison algorithms with various numbers of targets is investigated. Six tests are carried out with 4, 6, 8, 10, 12, 15 targets, in turn, and the size of the environment is 300 m × 300 m. There are 50 robots in the environment, and each robot can work 300 s. Experiments with the different initial positions of robots are implemented 400 times in every test.

**Figure 13:**
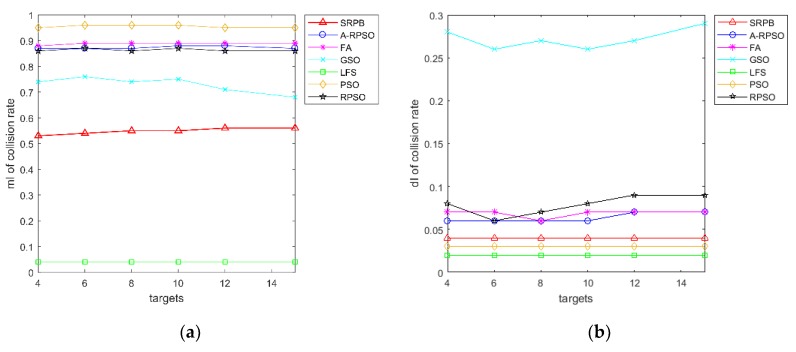
Collision rate of the strategies with various numbers of targets and 50 robots. (**a**) mI of collision rate; (**b**) dI of collision rate.

In Figure 13, the collision rate of different algorithms remains basically unchanged in a different number of targets. The results in part 5.5 show the collision rate of SRPB is 83% for 50 robots when the environment is 100 m × 100 m. In part 5.6, the collision rate of SRPB is 56% for 50 robots when the environment is 300 m × 300 m. We could infer that the collision rate is influenced by the environment and the population sizes. Once the environment and the population size are determined, the collision rate of algorithms does not vary with the number of targets. According to the collision rate, these strategies are sorted as LFS>SRPB>GSO>RPSO≈A-RPSO≈FA>PSO.

**Figure 14:**
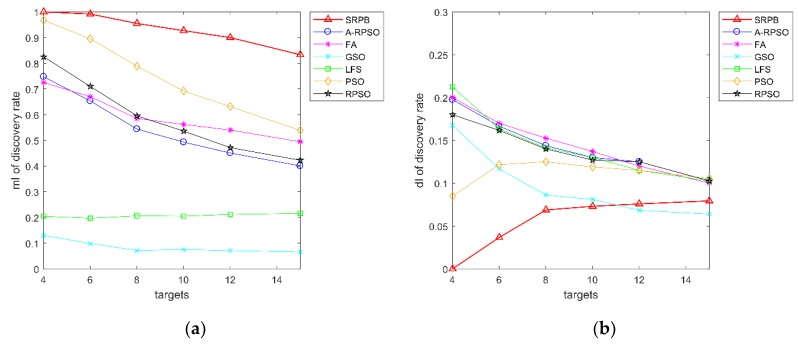
Discovery rate of the strategies. (**a**) mI of discovery rate; (**b**) dI of discovery rate.

The contrast curves of the discovery rate of the strategies are given in Figure 14. In the same environment, the average discovery rate of SRPB is greater than other strategies, and PSO is the suboptimum strategy. With the increase of targets, the average discovery rate decreases gradually. PSO, RPSO, A-RPSO, FA, GSO, FA, and LFS decline more sharply than SRPB. We could infer that the number of located sources is related to the number of robots. There are some speculations. In the ideal case, fifty robots could simultaneously find fifty targets in oriented search. Of course, it just suits the situation that a robot has found a target, and it cannot search the other targets. In the non-oriented search, the number of located sources is less than the number of robots because a source is located by a group of robots. In this paper, the maximum size of a group was four. It means that fifty robots can form twelve groups, at least. When the targets in the environment are in abundance, and the distribution of targets is not sparse, fifty robots in SRPB can find twelve targets, at least. As shown in Figure 14, fifty robots in SRPB find an average of 12.5 targets. Finally, according to the discovery rate, these algorithms can be sorted as SRPB>PSO>RPSO≈A-RPSO≈FA>LFS>GSO.

**Figure 15:**
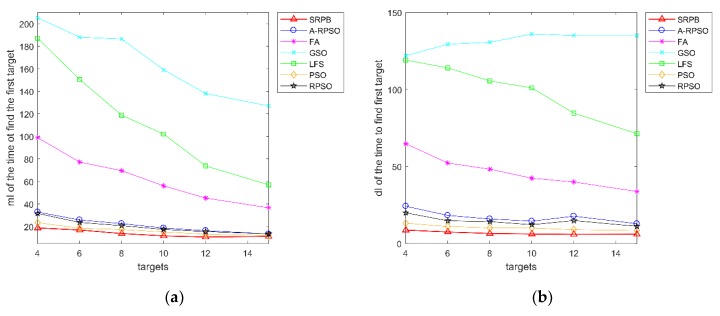

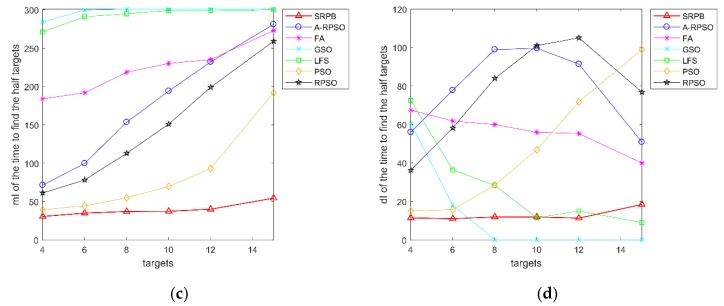
The time to find sources. (**a**) mI of the time to find the first target; (**b**) dI of the time to find the first target; (**c**) mI of the time to find the half targets; (**d**) dI of the time to find the half targets.

According to the time to find the first target, these algorithms can be sorted as SRPB≈PSO>RPSO A-RPSO>FA>LFS>GSO, and Figure 15c shows SRPB>PSO>RPSO>A-RPSO>FA>LFS≈GSO. Furthermore, Figure 15b,d show that SRPB is more stable than other algorithms because it has a low standard deviation. With the number of targets increasing, SRPB has more advantages than other algorithms in terms of the time to find the half targets. In Table 1, when the number of targets exceeds a certain value, robots in some algorithms can’t find the last target. It concludes that the number of located sources is related to the population size of robots.

All in all, the proposed algorithm can find targets as many as possible, and it has excellent stability, quick source seeking, and low collision rate. The overall performance of SRPB is better than PSO, RPSO, A-RPSO, GSO, FA, and LFS.

### 5.7. Practical Application Analysis

As mentioned before, the comparison reveals that the SRPB strategy has better performance than other algorithms. Some analyses are given to illustrate and analyze how to implement the strategy in a real robot.

Multi-source seeking is a significant problem. In reality, there are many applications about source seeking. For example, consider in the maritime rescue, there are several people with wireless transmitters for help. The autonomous unmanned aerial vehicle and unmanned surface vehicles can be used in this scenario to locate the positions of people. Since the radio signal is non-oriented, robots can locate the person with received signal strength taken at the robots’ position. Besides, the limited communication range will influence the cooperation, and the robot cannot be remotely controlled. Fuel consumption limits the working time. An unknown number of sources and unknown extrema make this task difficult. The method in this paper could be implemented in this situation. Each robot updates its velocity and position by Equations (29), (30), and stores a set of virtual match points. There are some assumptions. The width and length of the environment are *W*, *L*, respectively. The working time is *T*, and the number of robots is $N_{r}$. In this simulation, the computation complexity of the SRPB is $O(T\cdot N_{r}\cdot W\cdot L)$, and the space complexity is $O(N_{r}\cdot W\cdot L)$. In reality, each robot determines the motion by itself, and the computation complexity of a robot implementing the SRPB is O(W⋅L), and the space complexity is O(W⋅L). In a general control processor, such as ARM, the SRPB can be implemented. There is enough storage space to store the information of the environment because the number of virtual match points is small. Every robot equips with receiving antenna to receive the strength of the signal. In other types of sources, corresponding sensors are equipped in robots to receive the strength of signals. Besides, only the position and the corresponding signal strength are required to exchange with each other, so the information of communication is also small.

In conclusion, the strategy can be implemented in reality because all required aspects, including processor, communication, sensor, and scene, are met.

## 6. Conclusions

In this paper, we reviewed the target search algorithms and gave a classification. Aiming at the multiple weak sources seeking problem for swarm robots in an unknown environment, a model of the multi-target with different signals was given. Inspired from pedestrian behavior in subway/railway stations, a novel cooperative strategy, swarm robotic pedestrian behavior (SRPB), was proposed. It considered many realistic constraints, including limited communication range, limited working time, unknown sources, unknown extrema, the arbitrary initial location of robots, non-oriented search, and no central coordination. The robots’ trajectories from initial locations to the extrema showed that SRPB could effectively complete the task of multiple source seeking. The performance of the SRPB was evaluated in terms of average time to find the first, the half, and the last source, the number of located sources and the collision rate. Several experiments showed that SRPB had the highest efficiency and the best stability in all comparison strategies, and it had a low collision rate and a high number of located sources. Besides, numerous experiments demonstrated that the collision rate was related to the environment size and the number of robots, and the number of located sources was related to the number of robots. Finally, the analysis of how to implement this strategy, in reality, was given to support further research. 

## Figures and Tables

**Table 1:** mI and dI of the time to find the last target.

Number of Target	SRPB		A-RPSO		FA		GSO		LFS		PSO		RPSO	
	mI	dI	mI	dI	mI	dI	mI	dI	mI	dI	mI	dI	mI	dI
4	68.69	28.79	258.8	77.79	288.4	29.58	301	0	300.9	1.5	123.4	82.86	230.9	89.6
6	114.9	68.9	295.9	29.27	297.8	13.79	301	0	301	0	214.5	96.65	286.8	47.1
8	197.9	91.9	301	0	300	3.16	301	0	301	0	286.8	46.17	300	9.49
10	244	80.1	301	0	301	0	301	0	301	0	300	14.7	301	0
12	278	53.7	301	0	301	0	301	0	301	0	301	0	301	0
15	297.9	19.6	301	0	301	0	301	0	301	0	301	0	301	0
